# Edible quality of soft‐boiled chicken processing with chilled carcass was better than that of hot‐fresh carcass

**DOI:** 10.1002/fsn3.928

**Published:** 2019-01-28

**Authors:** Huhu Wang, Yue Qin, Jihao Li, Xinglian Xu, Guanghong Zhou

**Affiliations:** ^1^ Key Laboratory of Meat Products Processing MOA Nanjing Agricultural University Nanjing China; ^2^ Jiangsu Collaborative Innovation Center of Meat Production and Processing, Quality and Safety Control Nanjing Agricultural University Nanjing China

**Keywords:** chilling, edible quality, hot‐fresh, soft‐boiled chicken, yellow‐feathered broilers

## Abstract

Soft‐boiled chicken is widely popular with its flavor and texture. In a traditional view, the edible quality of soft‐boiled chicken producing with hot‐fresh carcass (without any chilled procedure after evisceration) was better than that of chilled carcass. Hot‐fresh groups with 1, 2, or 4 hr and chilled groups with 24, 48, or 60 hr were used to clarify the view in this study. The results indicated that no significant difference in hardness, springiness, cohesiveness of texture profiles and b* value of skin color was observed between each group, although the highest L* value was obtained in hot‐fresh 4 hr group. Higher contents of succinic acid were found in chilled groups when compared to that of hot‐fresh groups, but there was no difference in lactic acid and pH values. Lower contents of adenosine 5′‐monophosphate (AMP), guanosine 5′‐monophosphate (GMP), inosine and hypoxanthine, and higher inosine‐5′‐monophosphate (IMP) (especially for hot‐fresh 1 hr) were observed in hot‐fresh groups. In addition, although no difference in umami amino acids and bitter amino acid was observed between each tested group, higher amounts of Asp, Met, Ile, Leu, Tyr, and Arg were observed in chilled groups, especially for chilled 60 hr. The finding indicated that the traditional view was lack of scientific evidence, and chilled carcass was suitable for soft‐boiled chicken, substituting for the hot‐fresh carcass.

## 1 INTRODUCTION

The global production of chicken meat has increased from 73.1 million tons in 2008 to 90.2 million tons in 2017, especially for Asian countries. China has become the second most consumptions of chicken (FAS/USDA, 2017). The yellow‐feathered broilers, accounting for approximate 50% heads of chicken in China, were commonly consumed as a special product named soft‐boiled chicken, which has a distinctive flavor and texture (Gao et al., 2016; Jayasena, Jung, Alahakoon, et al., 2015; Jayasena, Jung, Bae, et al., 2015). Korean researcher demonstrated that yellow‐feathered chickens contained higher levels of inosine 5′‐monophosphate, betaine, and carnitine than white commercial broilers (Jayasena, Jung, Kim, et al., 2015). Moreover, the average price of yellow‐feathered broilers was much higher than white‐feathered broilers, such as Arbor Acres and Ross 308 (Zhang, Wang, Li, Wu, & Xu, 2016). Traditionally, the live yellow‐feathered broilers approved by the purchaser in wet‐markets were individually sold, and the carcasses were consumed by boiling or stewing. However, wet‐markets have been restricted in most cities of China since 2015 due to the outbreaks of animal influenza, and the traditional consumption pattern using hot‐fresh carcasses (not subjected to any chilled procedures) suffered from these restrictions. Soft‐boiled chicken is a very popular dish prepared by yellow‐feathered broilers, it is famous for its flavor and texture and simple preparation, consisting of boiling without seasoning until just cooked (Rui, Zhan, & Lin, 2008). The majority of consumers prefer to soft‐boil chicken processed with hot‐fresh carcasses rather than chilled carcasses, they believe that hot‐fresh chicken is more tender and flavor than any chilled chicken. However, the assumption was just a traditional view; no scientific data have refused it.

**Figure 1:**
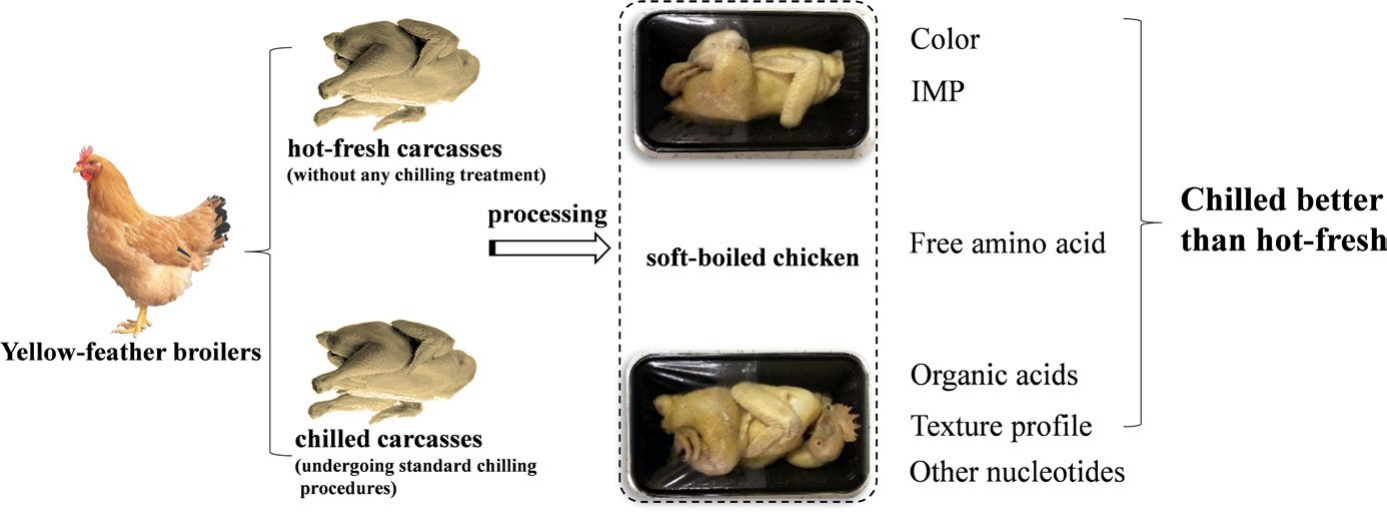
The Whole research proposal of this working

Several studies have addressed how post‐slaughter storage affects the edible quality raw and cooked meat. Generally, the concentrations of metabolites in broiler were varied during chilled storage, ATP, ADP, and AMP could be rapidly broken down, most of the potential ribose present in broiler meat was believed to be requirement for flavor formation (Aliani, Farmer, Kennedy, Moss, & Gordon, 2013); the breakdown of inosine‐5′‐monophosphate (IMP) can led to increases in inosine during chilled storage, and the overall water‐soluble flavor precursor profile could influence the sensory attributes (Williamson, Ryland, Suh, & Aliani, 2014). Using an electronic nose and ultra‐fast gas chromatography, Górska‐Horczyczak, Wojtasik‐Kalinowska, Guzek, Sun, and Wierzbicka (2017) observed that there were significant differences in the profile of volatile organic compounds of chilled meat during 1, 4, or 7 days storage. Van Ba, Oliveros, Park, Dashdorj, and Hwang (2017) have demonstrated that aging significantly reduced the shear force and increased the tenderness, flavor, and overall‐likability scores of meat. However, there is limited information on the effects of hot‐fresh and chilled carcasses on the texture and taste‐active compounds in soft‐boiled chicken. The aim of this study was to determine whether the texture and taste‐active compounds of soft‐boiled chicken prepared with hot‐fresh carcass was better than that of chilled carcass (Figure 1).

## 2 MATERIALS AND METHODS

### 2.1 Sample preparation

Thirty‐five Xueshan yellow‐feathered broilers (115 days old and approximate 1.1 kg weight of each carcass) were provided by the Lihua Food Co., Ltd., Changzhou, Jiangsu Province in China. The broilers were divided into two groups. (a) Fifteen chickens were removed immediately from slaughter line after evisceration and were not chilled with any treatments. The carcasses were stored at room temperature (20°C) for 1, 2, or 4 hr, and these treatments were defined as the hot‐fresh groups: whole carcass with bones and without viscera; (b) twenty chickens were subjected to a water‐chiller system for approximately 40 min and then stored at 4°C for 24, 48, or 60 hr. These treatments were defined as the chilled groups. The five chickens used for each experimental index served as experimental replicates. Hot‐fresh 1 and 2 hr were used to stimulate the time interval under traditional consumption pattern: purchase the broiler in wet‐markets and cook it immediately after being slaughtered. Hot‐fresh 4 hr usually simulated the scenarios: purchase the broiler at 06:30–07:30 a.m. and cooked it at 10:30–11:30 a.m. Chilled 24 and 48 hr were used to stimulate the time interval from broiler being slaughtered in large‐scale plants to cooking in kitchens at different area radius of meat consumption; chilled 60 hr was threshold of shelf‐life of chilled broiler under the current cold‐chain conditions.

The soft‐boiled chicken was processed by the traditional procedures. The raw carcasses were firstly cleaned the residual blood on the skin and lumen and then immediately transferred to ice water after immersing into the boiling water for 3 s, which could greatly shrink the skins. The carcasses were placed into boiling water for 18 min and finally were chilled with ice‐water for approximately 15 min. The right breast was used for texture profile analysis (TPA) and pH, and the left breast and thigh (1.5:1, w/w) were trimmed of visible fat and excess connective tissues and ground to homogeneity by a mincing machine (GM200; Retsch GmbH, Rheinische StraBe 36, 42781 Haan, Germany) for analysis of free amino acids, nucleotides, and organic acids.

### 2.2 Chemicals

Inosine 5′‐monophosphate disodium (IMP), guanosine 5′‐monophosphate (GMP), adenosine 5′‐monophosphate (AMP), hypoxanthine (Hx), and inosine (In) were purchased from Sigma‐Aldrich (Shanghai, China). Lactic acid, succinic acid, and 5‐sulfosalicylic acid dihydrate were obtained from Aladdin Chemical Reagent Co., Ltd. (Shanghai, China). A ninhydrin coloring solution kit for Hitachi and a MCl buffer L‐8500‐PH kit were purchased from Wako Pure Chemical Industries, Ltd., Japan. Perchloric acid, n‐hexane, methanol, potassium phosphate monobasic, ammonium phosphate dibasic, and the other common chemicals were obtained from Nanjing Chemical Reagent Co., Ltd.

### 2.3 Color measurement

The skin color of soft‐boiled broiler was measured using a chromameter (Minolta CR400, Konica Minolta Company, Tokyo, Japan). The average lightness (L*) and yellowness (b*) of skin color from the back, leg, and chest was chosen to represent the color of the whole product. A white color standard reference was used for instrument calibration.

### 2.4 Texture profile analysis (TPA)

Texture profile analysis was performed at room temperature according to the method described by Zheng et al. (2015) to assess the hardness, springiness, cohesiveness, chewiness, and resilience of samples using a texture analyzer (TA‐XT plus, Stable Micro Systems Ltd., UK). Cube‐shaped samples (10‐mm length, 10‐mm width, 10‐mm height) were removed from the cranial position of the right breast. A cylindrical probe (SMP P/50, flat bottom, diameter of 50 mm) and a double compression cycle were used to compress the samples to 50% of their original height. The TPA parameters were 5 g of force, a pre‐test speed of 3 mm/s, a test speed of 2 mm/s, and a post‐test speed of 3 mm/s. Exponent software (Exponent Stable Microsystem, version 5.1.2.0, Stable Microsystems Ltd., UK) was used to process the data.

### 2.5 Determination of pH

Five grams of sample was homogenized with 20 ml of distilled water at 10,000 g (three times, 15 s for each) using an Ultra‐Turrax T25 homogenizer (IKA, Staufen, Germany). The pH was determined with a pH meter (Mettler‐Toledo Instruments Co., Ltd., Zurich, Switzerland).

### 2.6 Determination of organic acids

The contents of organic acids were determined by high‐performance liquid chromatography (HPLC). Four grams of samples was homogenized and extracted with 20 ml of ultra‐pure water using an Ultra‐Turrax macerator (IKA, Staufen, Germany) in an ice bath. The homogenate was centrifuged at 12,000 *g* for 15 min at 4°C. The supernatant (25 μl) was passed through a 0.45‐μm membrane filter before HPLC analysis using an AtlantisT3 column (150 mm × 4.6 mm I.D., 5‐μm particle size) and a UV detector set at 214 nm, with the mobile phase of (NH_4_)_2_HPO_4_ (pH = 2.9, 5 g/L), 0.8 ml/min of flow rate, 20 min of running time. Each organic acid was identified and quantified by comparison of their retention times and peak areas to those of the corresponding standard.

### 2.7 Determination of nucleotides

The contents of nucleotides were determined using the method described by Liu, Xu, and Zhou (2007) with some modification. Four grams of samples was extracted with 20 ml of 5% (v/v) HClO_4_ using homogenization and centrifugation procedures similar to those outlined above (section Determination of organic acids). The pH of the supernatant was adjusted to 4.5 with potassium hydroxide, and then, the solution was diluted to 100 ml in a volumetric flask. Twenty microliters of supernatant was subjected to HPLC analysis after being passed through a 0.22‐μm membrane filter. An X‐Bridge C18 column (250 mm × 4.6 mm I.D., 5‐μm particle size) at a constant temperature of 25°C and a UV detector set at 254 nm were used. The retention times and peak areas of standards were used for quantification and identification of the nucleotides.

### 2.8 Determination of free amino acids

Four grams of samples was extracted using 3% (w/v) 5‐sulfosalicylic acid dihydrate. The homogenization and centrifugation procedures were conducted as described above (section Determination of organic acids). Four microliters of supernatant was removed and mixed with 2 ml of n‐hexane for 30 s. The aqueous phase was filtered through a 0.22‐μm membrane filter and analyzed with an amino acid autoanalyzer (L‐8900, Hitachi Co., Japan).

### 2.9 Statistical analysis

Five replicate carcasses for each treatment were performed for each index (*n* = 5). The results are shown as the means ± standard deviation. One‐way analysis of variance (ANOVA) and Duncan's new multiple range test were carried out to compare significant differences between results (*p* < 0.05) using the SAS 9.0 program (SAS Institute Inc., Cary, NC, USA).

## 3 RESULTS AND DISCUSSION

### 3.1 Color

The L* and b* values of the soft‐boiled chicken were shown in Table 1. There was no significant difference in the b* values among the treatments, and the L* values ranged from 70.46 to 74.67. The average L* value of the 1 hr hot‐fresh was significantly higher than those of others. Storage time affected the L* value, as the L* values of the hot‐fresh 1 hr and chilled 24 hr were higher than those of samples subjected to the same treatments but for longer periods. The skin color of the soft‐boiled chicken was used to represent the color of the whole product, since skin color is more important than meat color in the assessment of this product. Meanwhile, the meat colors showed no significant differences (data not shown). In addition, the product is typically sliced before serving, and the different parts including legs, chest, and back, are commonly served together. The L* values for all treatments were more than 70, and longer periods could result in lower L* values. Oxidative reactions of lipids and exudation of water occurred during storage, and these phenomena have been demonstrated to reduce L* values (Muela, Sañudo, Campo, Medel, & Beltrán, 2010; Vieira, Diaz, Martínez, & García‐Cachán, 2009). Moreover, although other studies showed that the b* values decrease during storage (Estrada‐Solis, Figueroa‐Rodriguez, Figueroa‐Sandoval, Hernandez‐Rosas, & Hernandez‐Cazares, 2016; Fernandes, de Alvarenga Freire, da Costa Carrer, & Trindade, 2013), no significant differences in the b* values were observed in our study. Consumers tend to prefer brighter and more yellow soft‐boiled chicken (Chen, Guo, Xu, Lu, & Zhang, 2014), and a brighter appearance to the soft‐boiled chicken can increase the consumer's desire to eat it.

**Table 1:** Skin color of chicken that was soft‐boiled after being subjected to various storage treatments

Groups	L* value	b* value
Hot‐fresh 1 hr	74.67 ± 2.51^a^	28.68 ± 5.09
Hot‐fresh 2 hr	71.44 ± 2.80^b^	27.61 ± 5.90
Hot‐fresh 4 hr	70.46 ± 3.09^b^	26.67 ± 3.08
Chilled 24 hr	72.29 ± 2.45^ab^	28.67 ± 4.27
Chilled 48 hr	72.16 ± 4.34^ab^	29.74 ± 3.80
Chilled 60 hr	71.31 ± 3.50^b^	29.74 ± 3.27

*Note*

Values are means ± *SD*,* n* = 5. Values in columns having different superscript letters are significantly different.

### 3.2 Texture profile analysis (TPA)

Texture is quite important for determining food quality and directly affects acceptance by consumers (Piqueras‐Fiszman & Spence, 2012). Soft‐boiled chicken is desired for its smooth and elastic texture, which may be ascribed to high amounts of total collagen but less soluble collagen, and is a crucial element for assessing the overall quality. The texture profiles of the soft‐boiled chickens are analyzed in Table 2. In general, there was no significant difference in hardness among the samples subjected to the various treatments. Similar trends were seen in the cohesiveness and springiness values for the hot fresh and chilled treatments. The chewiness values ranged from 938 to 1,446 g, and the hot‐fresh 4 hr and chilled 60 hr resulted in the highest chewiness values among their respective groups.

**Table 2:** Texture profile analysis of chicken that was soft‐boiled after being subjected to various storage treatments

Groups	Hardness (g)	Springiness	Cohesiveness	Chewiness (g)	Resilience
Hot‐fresh 1 hr	3,543 ± 949.64	0.553 ± 0.044	0.480 ± 0.031	938 ± 264.63^d^	0.185 ± 0.015^b^
Hot‐fresh 2 hr	3,889 ± 841.39	0.560 ± 0.060	0.493 ± 0.039	1,049 ± 183.32^cd^	0.197 ± 0.027^ab^
Hot‐fresh 4 hr	4,232 ± 799.47	0.578 ± 0.013	0.490 ± 0.048	1,391 ± 124.93^ab^	0.196 ± 0.014^ab^
Chilled 24 hr	4,193 ± 551.06	0.588 ± 0.050	0.532 ± 0.087	1,250 ± 200.90^abc^	0.222 ± 0.044^a^
Chilled 48 hr	3,841 ± 1,259.67	0.604 ± 0.033	0.499 ± 0.033	1,113 ± 124.10^b^	0.202 ± 0.025^ab^
Chilled 60 hr	4,486 ± 434.70	0.605 ± 0.045	0.535 ± 0.050	1,446 ± 119.47^a^	0.220 ± 0.019^a^

*Note*

Values are means ± *SD*,* n* = 5. Values in columns having different superscript letters are significantly different.

### 3.3 Organic acids and pH

The levels of the succinic acid and lactic acid are measured in Table 3. There were significant differences among the levels of succinic acid within the hot‐fresh treatments. The highest content of succinic acid was observed in the chilled‐60 hr treatment, reaching 1,280 mg/100 g, which was significant higher than that of the other groups except those chilled for 24 hr. Succinic acid is an important intermediate in the tricarboxylic acid cycle and has been reported to be one of the main taste‐active components in food, such as clam, where it can reach the levels of 800 mg/100 g and contribute a unique sour and umami taste. Succinic acid has been reported at levels of only 5–12 mg/100 g in cooked duck meat (Dai et al., 2011). In this study, the succinic acid contents ranged from 899.42 to 2,032.17 mg/100 g, which are much higher in levels than those reported in previous studies. Because of the role in the tricarboxylic acid cycle, the amount of succinic acid depends on the equilibrium between its formation and consumption. In this study, decreases in the succinic acid content might result from its conversion to other compounds during aging, while the increases observed for the final storage time points might be due to the accumulation of compounds that can be converted to succinic acid in the tricarboxylic acid cycle.

**Table 3:** Organic acids and pH values of chicken that was soft‐boiled after being subjected to various storage treatments

Groups	Succinic acid	Lactic acid	pH
Hot‐fresh 1 hr	1,280 ± 168.36^cd^	844 ± 159.11	5.93 ± 0.22
Hot‐fresh 2 hr	1,160 ± 234.34^cd^	840 ± 140.87	5.97 ± 0.07
Hot‐fresh 4 hr	899 ± 248.63^d^	1,183 ± 258.10	5.90 ± 0.16
Chilled 24 hr	1,710 ± 160.61^ab^	975 ± 154.49	5.99 ± 0.09
Chilled 48 hr	1,432 ± 408.46^bc^	873 ± 165.61	5.97 ± 0.07
Chilled 60 hr	2,032 ± 308.90^a^	1,099 ± 324.34	5.99 ± 0.11

*Note*

Contents of organic acids are in mg/100 g on the basis of dry matter and are expressed as the means ± *SD*,* n* = 5. Means with different superscript letters in the same column are significantly different, *p* < 0.05 (Duncan's multiple range test).

In this study, the content of lactic acid in the hot‐fresh 4 hr was significantly higher than other hot‐fresh groups. The content of lactic acid also increased at the end of chilled storage period. This increase might be due to the accumulation of lactic acid during postmortem aging. Generally, organic acids such as succinic acid and lactic acid have a sour taste (in an acidic environment) and sharply stimulate the tongue. Moreover, lactic acid is the most active ion, and its omission from a model broth system was perceived by 83% of the assessors in a sensory triangle test (Cerny & Grosch, 1992). However, these acids do not present the same taste at neutral pH or contribute to the sour taste when the pH of the beef broth is approximately 6.0 (England, Matarneh, Scheffler, Wachet, & Gerrard, 2014). The pH values of the samples in most groups ranged from 5.90 to 5.99, no difference was observed among these treatments, and these organic acids might have few impacts on sour flavor.

### 3.4 Nucleotides

The concentrations of nucleotides are shown in Table 4. IMP was the most abundant product of ATP breakdown in all groups and was the highest in hot‐fresh 1 hr group, which was attributed to high concentrations in raw chicken. The GMP, AMP, inosine, and Hx tended to increase during hot‐fresh storage. Similar trends were seen for these nucleotides in chilled treatments. The taste thresholds of IMP, AMP, and GMP are 25, 50, and 12.5 mg/100 g, respectively. As shown in Table 4, the concentrations of IMP were well above its taste threshold, but the concentrations of AMP and GMP were below the threshold, which may result from their migration from the meat into the broth, and the speculation has been confirmed in stewed chicken and goat meat (Madruga, Elmore, Oruna‐Concha, Balagiannis, & Mottram, 2010; Qi et al., 2018). Therefore, IMP might be the main contributor to the umami taste, while AMP and GMP make auxiliary contributions. In a previous study, inosine was found to generate a bitter taste, and Hx did not contribute to any taste response (Mateo & Zumalacárregui, 1996). This study found that Hx and inosine were present at low concentrations in samples stored for short periods, but increased linearly and steadily in concentration throughout storage, whereas the concentration of IMP decreased with increasing storage time. The trends in the contents of IMP, inosine, Hx, and AMP in the hot‐fresh and chilled treatment groups were consistent with reports showing that IMP is the predominant nucleotide, although its content decreases during storage, while those of inosine and Hx increase with storage time (Koutsidis et al., 2008).

**Table 4:** Nucleotides of chicken that was soft‐boiled after being subjected to various storage treatments

Groups	IMP^3^	AMP	GMP	Inosine	Hx
Hot‐fresh 1 hr	134.63 ± 22.68^a^	2.430 ± 1.112^d^	3.047 ± 0.588^d^	14.18 ± 1.88^e^	2.270 ± 0.240^b^
Hot‐fresh 2 hr	101.01 ± 17.94^bc^	2.493 ± 0.395^d^	6.693 ± 1.210^bc^	19.45 ± 1.42^cde^	3.190 ± 0.425^b^
Hot‐fresh 4 hr	93.25 ± 13.80^bcd^	3.608 ± 0.113^d^	6.427 ± 0.434^c^	17.83 ± 6.89^de^	3.850 ± 0.066^b^
Chilled 24 hr	90.81 ± 10.18^bcd^	6.609 ± 3.166^ab^	6.246 ± 1.013^c^	20.23 ± 2.69^cde^	5.851 ± 1.410^a^
Chilled 48 hr	83.21 ± 13.22^bcde^	8.563 ± 0.336^ab^	7.183 ± 1.033^abc^	32.54 ± 5.94^b^	6.362 ± 1.154^a^
Chilled 60 hr	86.45 ± 14.64^bcd^	9.845 ± 1.991^a^	8.750 ± 1.632^a^	28.36 ± 8.59^bc^	6.810 ± 1.470^a^

*Notes*

Nucleotide levels are in mg/100 g on the basis of dry matter and are expressed as the means ± *SD*,* n* = 5. Means with different superscript letters in the same column are significantly different, *p* < 0.05 (Duncan's multiple range test).

AMP: adenosine 5′‐monophosphate; GMP: 5′‐guanosine monophosphate; Hx: hypoxanthine; IMP: 5′‐inosinic acid.

### 3.5 Free amino acids

The levels of free amino acids were measured (Table 5). The total FAA concentration was close to values of Japanese yellow‐feathered chicken meat (Rikimaru & Takahashi, 2010), but was greatly higher than duck mea (Wang et al., 2016). The most abundant free amino acid detected was lysine (Lys), which constituted approximately 40%–60% of the total amino acids, but it has no taste. The least abundant was cysteine (Cys), which was usually present at levels below 1 mg/100 g. There was no significant difference among any of the free amino acid levels in the 3 hot‐fresh treatments with the exception of the serine (Ser) content, which could contribute flavor and was significantly lower in the hot‐fresh 2 hr treatment group. The concentrations of almost all the free amino acids increased with increasing chilled storage. The content of isoleucine (Ile) also increased, and significant differences were observed between the chilled‐24 hr and chilled‐60 hr groups. Similar trends were observed for tyrosine (Tyr) and arginine (Arg), and their contents in the chilled‐48 hr and chilled‐60 hr groups were significantly higher than those in the chilled 24 hr.

**Table 5:** Free amino acid contents of chicken that was soft‐boiled after being subjected to various storage treatments

Chemicals	Hot‐fresh 1 hr	Hot‐fresh 2 hr	Hot‐fresh 4 hr	Chilled 24 hr	Chilled 48 hr	Chilled 60 hr
Asp	3.48 ± 0.51^c^	4.79 ± 1.73^bc^	3.48 ± 0.98^c^	3.95 ± 0.53^bc^	5.15 ± 0.60^a^	5.50 ± 0.53^a^
Thr	35.44 ± 11.04^a^	27.47 ± 2.34^ab^	39.69 ± 11.00^a^	19.03 ± 3.87^b^	33.51 ± 10.44^ab^	32.59 ± 2.03^ab^
Ser	15.16 ± 3.88^ab^	11.37 ± 2.38^b^	14.82 ± 1.93^ab^	13.62 ± 1.63^ab^	15.09 ± 1.78^ab^	12.11 ± 0.98^b^
Glu	29.69 ± 8.60^a^	30.00 ± 3.19^a^	26.53 ± 6.39^ab^	25.85 ± 2.48^b^	33.66 ± 4.62^a^	32.08 ± 6.60^a^
Gly	10.66 ± 3.00^ab^	11.63 ± 1.45^a^	9.54 ± 0.60^ab^	8.91 ± 0.49^b^	9.75 ± 1.23^ab^	10.35 ± 1.21^ab^
Ala	21.57 ± 1.94^ab^	19.36 ± 6.49^b^	25.35 ± 5.97^a^	21.32 ± 3.58^ab^	22.47 ± 2.77^ab^	21.56 ± 1.11^ab^
Cys	1.01 ± 0.11^a^	1.033 ± 0.311^a^	1.02 ± 0.15^a^	0.62 ± 0.08^b^	0.81 ± 0.14^ab^	0.80 ± 0.23^ab^
Val	4.21 ± 0.15^c^	4.04 ± 1.17^c^	4.05 ± 0.15^c^	4.66 ± 0.87^bc^	5.60 ± 0.64^b^	6.03 ± 0.77^ab^
Met	1.53 ± 0.31^d^	1.73 ± 0.32^d^	1.79 ± 0.26^cd^	2.54 ± 0.47^b^	3.24 ± 0.76^a^	2.67 ± 0.36^a^
Ile	2.27 ± 0.51^b^	2.42 ± 0.76^b^	2.71 ± 0.44^b^	3.46 ± 0.46^a^	4.09 ± 0.60^a^	4.56 ± 1.25^a^
Leu	5.10 ± 0.84^b^	4.94 ± 0.60^b^	5.92 ± 0.80^b^	7.73 ± 1.04^a^	9.18 ± 1.34^a^	8.51 ± 1.32^a^
Tyr	2.79 ± 0.35^c^	3.17 ± 0.48^c^	3.24 ± 0.52^c^	5.23 ± 0.60^b^	6.56 ± 0.77^a^	6.85 ± 0.70^a^
Phe	4.04 ± 0.23^b^	4.27 ± 0.67^b^	4.59 ± 0.64^b^	6.64 ± 0.56^a^	7.15 ± 0.79^a^	7.50 ± 0.97^a^
Lys	137.40 ± 23.22	145.74 ± 26.83	145.34 ± 12.76	137.68 ± 11.97	131.78 ± 4.55	132.68 ± 8.82
Arg	5.97 ± 0.46^cd^	4.97 ± 0.59^d^	6.65 ± 0.52^c^	7.95 ± 0.32^b^	9.81 ± 1.06^a^	9.72 ± 0.53^a^
Pro	8.02 ± 0.65^ab^	7.48 ± 1.46^b^	8.23 ± 0.97^ab^	7.79 ± 1.07^b^	8.91 ± 0.76^ab^	9.64 ± 0.85^ab^
UAA^3^	29.71 ± 6.65	34.78 ± 4.82	30.02 ± 7.13	29.80 ± 2.75	38.81 ± 5.10	37.58 ± 6.84
SAA	86.14 ± 19.17^ab^	76.25 ± 11.09^ab^	85.04 ± 22.67^ab^	65.61 ± 2.07^b^	87.75 ± 16.19^a^	86.23 ± 3.66^ab^
BAA	162.80 ± 25.67	189.92 ± 41.29	174.47 ± 14.50	174.41 ± 8.51	174.65 ± 14.35	178.49 ± 14.32
Total	285.66 ± 28.71^a^	301.98 ± 34.26^ab^	295.64 ± 19.68^ab^	271.72 ± 22.55^b^	292.18 ± 15.73^a^	303.09 ± 19.73^ab^

*Notes*

Contents of the free amino acids are in mg/100 g on the basis of dry matter and are expressed as the means ± *SD*,* n* = 5. Means with different superscript letters in the same row are significantly different.

BAA: bitter amino acid; SAA: sweet amino acids; UAA: umami amino acids.

Asp and Glu are important contributors to the umami flavor. Meanwhile, Cys is the origin of S‐containing compounds (Qi, Liu, Zhou, & Xu, 2017). In this study, only Lys was present at levels above its taste threshold (50 mg/100 g). However, free amino acids have strong impacts on taste even when present in low concentrations because of their low taste threshold values. At low concentrations, certain free amino acids would have a synergistic effect on the umami taste. Likewise, the sweet amino acids synergistically interact with IMP, a contributor to the umami flavor, and as a result, the sweetness is increased by the presence of IMP (Kawai, Okiyama, & Ueda, 2002).

The balance between the formation and degradation of free amino acids ultimately controls their contents. Increases in the contents of free amino acids are primarily due to the activity of aminopeptidases, which decompose protein and peptides, while decreases are principally related to the formation of volatile components from free amino acids. Studies on cooked duck and ham showed that Glu and Ala were the most abundant free amino acids, and they were generally present at concentrations above 100 mg/100 g (Liu et al., 2007; Martın, Antequera, Ventanas, Benıtez‐Donoso, & Córdoba, 2001). A number of studies have reported increases in the contents of free amino acids during postmortem conditioning, which substantially impact the flavor (Calkins & Hodgen, 2007; Koutsidis et al., 2008). In beef broth, the elevated amounts of Glu, Asp, Lys, and Met after conditioning great enhanced its flavor (Pereira‐Lima, Ordoñez, García de Fernando, & Cambero, 2000). The significant development of taste intensity in salted meat is correlated with the increase in the content of free amino acids during aging (Rabie, Simon‐Sarkadi, Siliha, El‐seedy, & El Badawy, 2009). In this study, the concentrations of Glu and Ala were below their taste thresholds (30 mg/100 g and 60 mg/100 g, respectively). This difference might be due to the different cooking processes and low handling temperature throughout the processes.

## 4 CONCLUSIONS

The storage time for raw chicken before cooking impacted the texture and taste‐active compounds of soft‐boiled chicken. Chilled treatments resulted in similar texture and taste parameters as those observed when using chickens stored hot‐fresh for 4 hr. Even though chickens that were stored hot‐fresh for 1 hr had the highest IMP and L* values, the treatment resulted in a less desirable texture than was achieved with chilled treatments. In other words, chickens that were stored hot‐fresh for 1 hr might afford dishes with slightly more “umami” taste but with poorer mouth‐feel. Therefore, this study showed that it is feasible to use chilled yellow‐feathered carcasses to prepare soft‐boiled chicken that is similar in texture and taste‐active compounds to what can be achieved by using freshly slaughtered (hot fresh) chicken. In addition, for the industrial production of soft‐boiled chicken, the chilled chicken can be used to prepare the dish on a large scale and promote the commercial production of this dish.

## CONFLICT OF INTEREST

The authors declare that they do not have any conflict of interest.

## ETHICAL STATEMENT

This study does not involve any human or animal testing.
